# Fluorometric combination analysis of conformational changes in urea-induced unfolding of soybean protein isolates and their complexes with casein

**DOI:** 10.1371/journal.pone.0319864

**Published:** 2025-03-31

**Authors:** Yangchao Gao, Yuchuan Li, Yifan Yang, Hongying He, Yanqiong Li, Shuhui Yu, Haiying Wang, Yuyu Zhang, He Liu

**Affiliations:** Yunnan Characteristic Resource Plants Intelligent Agriculture Engineering Center, College of Agriculture and Life Science, Kunming University, Kunming, China; University of Waterloo, CANADA

## Abstract

Soybean proteins isolates (SPIs) could misfold to form amorphous aggregates after unfolding due to processing conditions, leading to a decrease in their solubility, and casein might be able to block the formation of amorphous aggregates. The turbidity curves showed that SPIs/7S/11S could maintain a soluble state in the presence of casein. The folding and unfolding processes of SPIs are not a single-step event. Fluorescence phase diagram method was utilized to analyze the unfolding process of SPIs and SPI-casein complexes. The results showed that there was one intermediate (molten globule, MG) in the unfolding process of SPIs, while there were two intermediates (dimer or trimer intermediates and MG) in that of SPI-casein complexes. Further attempts were made to explain the conformational changes of SPIs when binding with casein. The surface hydrophobicity index indicated that the intermediate with strong hydrophobicity was exposed when induced by 2 mol/L urea. The fluorescence quenching experiments using KI as a quencher showed that the *K*_*sv*_ of SPIs and 11S and their complexes with casein all reached peak values when urea concentration was 2 mol/L, while *K*_*sv*_ values of 7S and 7S–casein complex peaked at urea concentration 8mol/L and 6mol/L, respectively. Besides, *K*_*sv*_ and *f*_*m*_ changing trends of 7S–casein complex was similar to that of SPIs–casein. Based on the above results, it could be inferred that the interactions between SPIs and casein mainly occurs in 7S. The molecular docking of 7S chains from SPIs and casein showed that SPIs could form a “pocket” conformation to “hold” the casein chain. These findings provide new perspectives for understanding the mechanism of interaction between soy proteins and casein, and may provide a basis for research on the functionality of proteins in the food industry. Note: *K*_*sv*_ is the quenching constant of the quenching agent, and *f*_*m*_ is the quenching rate.

## Introduction

Soybean protein isolate (SPI) is widely used in beverages, meat products, and baby food due to its good processing properties, such as emulsification, gel formation, high nutritional value, and physiological activity. However, the operating conditions of food processing often lead to the loss of SPIs solubility, which directly results in turbidity or precipitation in food systems, especially in some high protein food systems, making it a major challenge for commercial applications [1,2]. Under the influence of processing fields, including high-temperature sterilization and high pH, SPIs undergo structural unfolding followed by refolding. These processes could change their spatial structure and/or interactions, exposing residues like hydrophobic regions and sulfhydryl groups. These changes, in turn, facilitate cross-linking between protein molecules, ultimately leading to the formation of various aggregates with differing shapes and sizes, ranging from microgels and amorphous aggregates to fibrillar aggregates [3]. Among them, the formation of amorphous aggregates is considered to be associated with the reduced solubility of SPIs. Due to the complexity of protein structures, the unfolding process is far from “single-step” complete unfolding, giving rise to the appearance of different partially folded intermediates. Therefore, exploring the conformational changes of intermediates during protein unfolding is significant for understanding the protein folding process.

Currently, the most commonly studied methods for inhibiting protein aggregation and increasing solubility mainly include the addition of small molecule chemicals or adjusting the hydrophobicity or charge of proteins. Yi et al. [4] found that adding tannic acid could stabilize the protein system and prevent its aggregation. Wang et al. [2] inhibited the thermal aggregation of soy globular proteins by adding phytates to increase the surface negtive charge of proteins. In addition, there have been reports on the use of molecular chaperones to enhance the solubility of protein solutions. Yan et al. [5] reported the inhibitory effect of ellagic acid on the heat-induced amyloid-like aggregation of ovalbumin. Hartl et al. [6] found that the pathway of amorphous aggregate formation through intermolecular interactions could be effectively blocked by molecular chaperones. In food systems, casein has also been reported to act as a molecular chaperone-like substance. Singh et al. [7] found that inclusion of caseins caused stabilization of whey proteins to UHT processing. In addition to serving as an inhibitor of protein aggregation and improving solubility, casein could also improve the nutritional value of the system, providing new insights for researchers. However, there are few reports on the molecular chaperone-like regulatory effects of casein on the formation of plant globular protein aggregates, and there is a lack of in-depth and systematic research. Previous studies in our laboratory have shown that the addition of casein could reduce the turbidity and improve the solubility of SPIs under high temperature (100 °C) and certain pH (5–7) treatment. Therefore, we speculated that the addition of casein may have blocked the formation of amorphous aggregates of SPIs.

Thus, in this study, the turbidity and different fluorometric methods were utilized to explore preliminarily the possible mechanism of casein blocking amorphous aggregates formation of SPIs, so as to provide new perspectives for understanding the mechanism of interaction between SPIs and casein, and innovate techniques to improve the stability of SPIs during food processing.

## Materials and methods

### Materials

Defatted soybean flakes were purchased from Shandong Wonderful Biotech Co., LTD. Casein (analytical reagent grade) was purchased from Tianjin Damao Chemical Reagent factory. All other chemicals were of analytical reagent grade.

### Preparation of SPIs, 7S and 11S samples

Soybean protein isolates (SPIs) were extracted as described by Lv, Yang, and Guo (2009) without any modification [8]. Defatted soybean flakes were mixed with water in the ratio of 1:15, and pH was adjusted to 8.0 with 6.0 M NaOH. After extraction at room temperature for 1 h, the mixture was centrifuged at 4000 rpm for 30 min. The supernatant was adjusted to 4.5 with 6M HCl, and then centrifuged again (4,000 rpm, 20 min) after 30min of resting, the precipitate was washed with distilled water for three times, and the precipitate was re-dissolved and the pH was adjusted to 7.0 with NaOH, and the resulting solution was lyophilized as SPIs.

7S and 11S were prepared according to the method of Clara Sze [9] with slight modification. Briefly, defatted soybean flakes were extracted with 15 volumes of distilled water adjusted to pH 8.0 with 6.0 M NaOH at room temperature for 1.5 h. The slurry was filtered and the filtrate was centrifuged (4,000 rpm, 20 min), and dry sodium bisulfite (0.98 g/L) was added to the supernatant. The pH of the mixture was adjusted to 5.4 with 6.0 M HCl, and the precipitate (11S) was removed by centrifugation (4,000 rpm, 5 min, 4°C). The precipitate was washed five times with distilled water, suspended in a minimum amount of distilled water, dialyzed and lyophilized for further use. All subsequent steps were performed at 4°C. The pH of the supernatant was adjusted 4.5 with 6.0 M HCl, the mixture was allowed to stand overnight and then centrifuged (4,000 rpm, 20 min, 4°C), and the precipitate (7S) was washed for 5 times with distilled water, then suspended in a minimum amount of distilled water, pH adjusted to 7.2 with 6.0 M NaOH, dialyzed and lyophilized for further use.

### Preparation of unfolded protein samples

Unfolded protein samples were prepared by dissolving 100 mg SPIs into 100 mL buffer with pH 7.0 and ionic strength 0.2 mol/L, which favored amorphous aggregates formation. The mixture was stirred for 5 h to dissolve the protein completely, and then urea was added to the buffer so that the urea final concentration was 0, 1, 2, 4, 6, and 8 mol/L. The solutions were incubated at 4 °C for 40 h to reach equilibrium.

Unfolded protein–casein complex samples were prepared by dissolving 50 mg SPIs and 50 mg casein into 100 mL buffer with pH 7.0 and ionic strength 0.2 mol/L, which favored amorphous aggregates formation. The mixture was stirred for 5 h to dissolve the protein completely, then urea was added to the buffer so that the urea final concentration was 0, 1, 2, 4, 6, and 8 mol/L. The solutions were incubated at 4 °C for 40 h to reach equilibrium.

### Turbidity measurements

Dissolve the protein samples or protein complexes samples with a ratio of 1:1 into a buffer, which favored the formation of amorphous aggregates (pH 7.0, ionic strength of 0.2 mol/L), adjusting the final protein concentration to 1 mg/mL. The absorbance (A) of protein samples or protein complexes samples (25°C) were measured at 540 nm [10] using a 1 cm pathlength glass cuvettes by a UV-1500 spectrophotometer (Macylab Instrument, Shanghai, China). Milli-Q water was used to calibrate for 0% absorptance. The absorbance value was defined as turbidity. The turbidity was measured for 120 min to draw the turbidity curve of all the protein solutions. All experiments were performed in triplicate.

### Intrinsic fluorescence spectra and fluorescence phase diagrams of the unfolding process

The intrinsic fluorescence emission spectra of protein solutions with different degrees of denaturation were recorded, and fluorescence phase diagrams were plotted in accordance with the fluorescence intensities *I*_320_ and *I*_365_. Instrument parameters were as follows: excitation wavelength of 295 nm, excitation slit and emission slit width of 10 nm, scanning range of 280–500 nm, and scanning speed of 600 nm/min [11].

### Determination of the surface hydrophobicity index of the unfolding process

The determination of surface hydrophobicity index was performed by the 1-Anilino-8-naphthalene-sulfonate (ANS) fluorescent probe method. The protein samples to be measured were diluted to 0.2, 0.4, 0.6, 0.8, and 1 mg/mL with unified buffer solution. About 4.5 mL of protein solutions of different concentrations were taken and mixed well with 0.5 mL of 1.25 × 10^ − 3^ mol/L ANS solution at room temperature, and then the reaction solution was kept away from light for 2 h, after which the fluorescence intensity *I* of the reaction solution was measured. Taking the protein concentration as the abscissa and the fluorescence intensity as the ordinate, the slope of the fitting line obtained was the surface hydrophobicity index *S*_*0*_ of the sample measured. Instrument parameters were as follows: excitation wavelength and emission wavelength were 390 and 470 nm, respectively, and the slit width was 5 nm.

#### Fluorescence quenching indexes of urea-treated unfolded protein samples.

Acrylamide and KI were selected as fluorescence quenching agents of SPIs and SPI–casein complexes. In accordance with the methods of Lehrer [12] and Eftink [13], the quenching agent acrylamide was prepared using 0.1 mol/L phosphate buffer solution with a pH of 7.0 and added to denatured protein solution treated with different urea concentrations. The final concentration of protein was 1.0 mg/mL, and the concentration gradient of acrylamide was 0.0, 0.1, 0.2, 0.4, 0.5, and 1.0 mol/L; the measurements were conducted after 2 h of settlement at room temperature. After deducting the corresponding blank, the fluorescence intensity at the maximum emission peak was taken, and the quenching-related indexes *K*_*sv*_ and *f*_*m*_ were calculated in accordance with the diagram plotted to Stern–Volmer formula; the quenching agent potassium iodide was prepared using 0.1 mol/L phosphate buffer solution with a pH of 7.0 to a final concentration of 5 mol/L (containing 2.0 mmol/L Na_2_S_2_O_3_ as reducing agent to prevent the formation of I^3-^ ions) and added to denatured protein solution treated with different urea concentrations. The final concentration of protein was 1.0 mg/mL, and the concentration gradient of potassium iodide was 0.00, 0.02, 0.04, 0.06, and 0.08 mol/L; the measurements were conducted after 2 h of settlement at room temperature. After deducting the corresponding blank, the fluorescence intensity at the maximum emission peak was taken, and the quenching-related indexes *K*_*sv*_ and *f*_*m*_ were calculated in accordance with the diagram plotted to Stern–Volmer formula. Instrument indexes were as follows: excitation wavelength of 295 nm, excitation slit and emission slit width of 10 nm, scanning range of 300–400 nm, and scanning speed of 1200 nm/min.

The 0.1 mol/L phosphate buffer solution with pH 7.0 was used to prepare potassium iodide into a 5.0 mol/L solution, and then it was added into the protein solution with different degrees of denaturation. The final concentration of protein was 1.0 mg/mL, and the concentration of potassium iodide was 0.00, 0.02, 0.04, 0.06, and 0.08 mol/L, respectively. After being placed for 2 h, spectral determination was conducted. After deducting the corresponding blank, the fluorescence intensity was taken at the maximum emission peak and plotted in accordance with the Stern–Volmer formula; then, the quenching related indexes *K*_*sv*_ and *f*_*m*_ were calculated. Instrument indexes were as follows: excitation wavelength of 295 nm, excitation slit width and emission slit width of 10 nm, scanning range of 300–400 nm, scanning speed of 1200 nm/min.

### Protein molecular docking and image processing

The online molecular docking tool ClusPro (https://cluspro.bu.edu/login.php) was used to dock the SPI (PDB: 1IPK) to the casein molecule (PDB: 5WO2) between different chains separately [14], and the docking results were saved; images were analyzed and exported using Pymol.

### Statistical analysis

The means and standard deviations for each treatment group in all experiments were calculated. The significance of differences between the means was evaluated by Scheffé’s test after two-way ANOVA by using SPSS (SPSS, Inc., Chicago, IL). Differences of *p* <  0.05 were considered statistically significant.

## Results and discussion

### Turbidity curves of SPIs/7S/11S and their casein complexes

**Fig 1:**
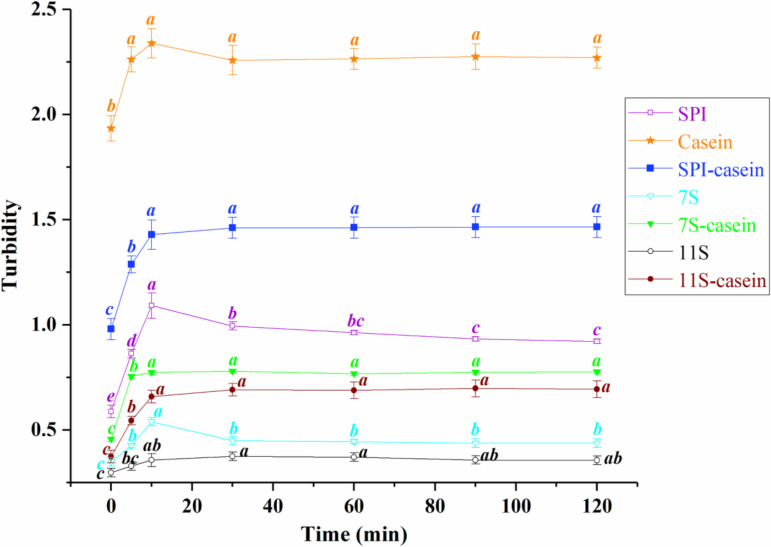
The turbidity changes of SPIs/7S/11S and their casein complexes samples as a function of time. Different letters in the same curve indicate significant difference between data (*p < *0.05).

Turbidity measurement serves as an intuitive and convenient method for characterizing interactions between proteins, and turbidity changes at different time could reflect the interaction changes in proteins [15]. As shown in Fig 1, with the extension of the dissolution time, the turbidity of SPIs solution showed a trend of increasing and then decreasing. The turbidity reached a maximum of 1.092 ± 0.06 at 10 minutes of dissolution, and then reduced to 0.995 ± 0.02 at 30 minutes (*p < *0.05). The turbidity of casein solution experienced a increase from 0 to 5 minutes to 2.262 ± 0.06, and then reached a stable state, the difference between the remained turbidity values was not significant (*p > *0.05). In the case of SPI-casein complexes, the turbidity values showed an increasing and then stabilizing trend. At 10 minutes of interaction, the turbidity value of the complexes increased to 1.429 ±  0.07, and the differences among the remained turbidity values were not significant (*p < *0.05).

The main component of SPIs is globulin, of which 7S (β-conglycinin) and 11S (glycinin) account for about 80% [16]. Thus, we also performed turbidity analyses on them and their casein complexes, respectively. As shown in the figure, it appeared that 7S showed a turbidity trend of increasing and then decreasing, which was similar to that of SPIs, and the 7S-casein complex also shows a trend which was similar to that of SPI-casein. However, the turbidity stabilization time (5 minutes) was earlier than that of SPI-casein complex (10 minutes). 11S-casein complexes showed the similar trend of turbidity change and turbidity stabilization time with SPI-casein complexes, which was increasing first and then stabilizing at 10 minutes.

Zhang et al. [17] found that protein forming amorphous aggregates or complexes with other proteins underwent a turbidity increase phase in the early stages. For soluble complexes, their turbidity slowly increased or remained unchanged after reaching the maximum, while proteins coagulation could to a decrease in turbidity. It could be inferred that as the dissolution and stirring time prolongs, the amorphous aggregates formed in the SPIs solution would undergo coagulation and precipitation, leading to a decrease in turbidity. In contrast, the interaction between SPIs and casein could keep them soluble by reducing the formation of amorphous aggregates, thus preventing coagulation and precipitation. The final turbidity of 7S and 11S also remained stable in the presence of casein, indicating that both of them could remain soluble in the presence of casein.

### Intrinsic fluorescence spectra and fluorescence phase diagrams of the unfolding process of soybean protein isolate induced by urea

Hirota et al. [18] believed that amorphous aggregates are formed during unfolding and refolding processes of globular proteins, and due to the complexity of the protein structures, different intermediates could be formed during refolding of globular proteins to fold into their final form. For example, when β-lactoglobulin was exposed to denaturing treatment conditions such as high temperature, its conformation underwent significant changes and the protein was in a partially denatured molten globule (MG) intermediate state, which aggregated into dimers or trimers intermediate state. The dimers or trimers intermediates could further fold to form primary aggregation forms such as amorphous aggregates [19]. Therefore, in order to investigate the presence and the number of intermediates in the unfolding process of SPIs, we investigated the unfolding process of SPIs with urea as the defolding agent.

Urea is a commonly used protein denaturant that affects the structural domains or even separates the domains from each other by breaking hydrogen bonds or inter- and intra-molecular weak interactions, thus affecting the entire protein conformation [20]. The formation process of protein aggregates can be preliminarily explored by treating soybean protein isolate solution with urea of different concentrations. The process of protein aggregate formation could be preliminary explored by treating SPIs at different urea concentrations.

**Fig 2:**
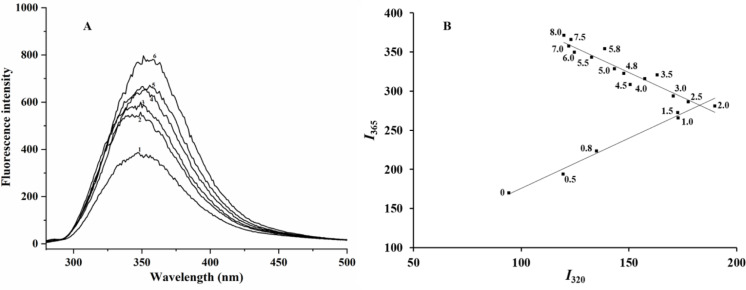
Intrinsic fluorescence spectra (A) and fluorescence phase diagram (B) of urea-induced unfolding of SPIs (Urea concentrations in the spectra: (1) 0M; (2) 1M; (3) 2M; (4) 4M; (5) 6M; (6) 8M).

The intrinsic fluorescence of proteins was mainly generated by the amino acid residues tryptophan (Trp), tyrosine (Tyr), and phenylalanine (Phe) in proteins. When 295 nm was used as the excitation wavelength, almost all the protein fluorescence originated from Trp; thus, the fluorescence changes of Trp residues were widely used in the study of conformational changes of protein. As shown in Fig 2A, with the gradual increase in urea concentration in the solution, the maximum fluorescence emission intensity of SPIs gradually increases, accompanied by the red shift of the maximum fluorescence emission peak. When the concentration of urea in the solution was in the range of 0.0–4.0 mol/L, the maximum fluorescence emission peak shifted from 343 nm to 356 nm, whereas the degree of red shift decreased to 353 nm when the concentration of urea reached 6.0–8.0 mol/L. That is, with the increase in urea concentration in the solution, the spatial structure of SPIs underwent a continuous and stable change, causing the tryptophan residues within their molecules to transfer gradually from the nonpolar region inside the molecule to the molecular surface and be exposed to the urea solution.

The fluorescence phase diagram method was used to investigate further the process of SPIs induced by urea. This method was first proposed by Burstein in 1971. Compared with other physicochemical methods for detecting structural changes in proteins, the fluorescence phase diagram method could provide more information about the protein unfolding process and may capture undetected partially folded intermediates [11]. The fluorescence phase diagram of urea-induced unfolding of SPIs consisted of two straight lines (Fig 2B), indicating that the urea denaturation process of SPIs was in accordance with the “three-state” model, that is, a partially folded intermediate state occurred in the unfolding process of the protein at the urea concentration between 2.0 and 2.5 M.

### Intrinsic fluorescence spectra and fluorescence phase diagrams of the unfolding process of SPIs–casein complexes induced by urea

**Fig 3:**
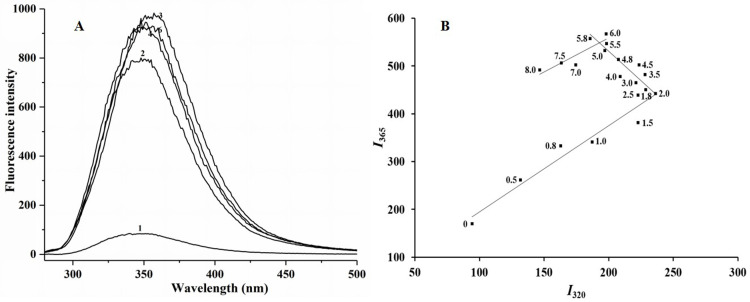
Intrinsic fluorescence spectra (A) and fluorescence phase diagram (B) of urea-induced unfolding of SPI-casein complexes (Urea concentrations in the spectra: (1) 0M; (2) 1M; (3) 2M; (4) 4M; (5) 6M; (6) 8M) (Note: 6M treated intrinsic fluorescence spectra was not shown because the signal was saturated).

As mentioned earlier, casein might block the formation of amorphous aggregates and maintain the turbidity of SPI-casein complexes. Therefore, the unfolding process of SPI-casein complexes were also investigated. During the unfolding process of protein complexes induced by urea (Fig 3A), the maximum fluorescence emission peak of the protein complexes was red-shifted with the gradual increase in the urea concentration in the solution. The maximum peak was red-shifted from 340 nm to 348 nm when the urea concentration in the solution increased from 0.0 mol/L to 2.0 mol/L, whereas the degree of red shift continued to increase to 351 nm when the urea concentration went up to 4.0 mol/L. The maximum peak position remained at 351 nm when the urea concentration increased from 4.0 mol/L to 8.0 mol/L. The fluorescence intensity of the maximum emission peak increased continuously when the urea concentration increased from 0.0 mol/L to 2.0 mol/L, but the fluorescence intensity decreased when the urea concentration reached 4.0 mol/L; then, a substantial increase occurred when urea concentration hit 6.0 mol/L, followed by a decrease at a urea concentration of 8.0 mol/L. Such repeated changes in fluorescence intensity with urea concentration indicated that the conformational changes during the unfolding of the urea-induced protein complexes were intricate, resulting in a changing microenvironment for the tryptophan residues.

The fluorescence phase diagram of the unfolding process of urea-induced protein complexes consists of three straight lines (Fig 3B), which indicate that the urea denaturation process of protein complexes was in accordance with a “four-state” model, that is, during the unfolding process of the protein, two partially folded intermediate states occurred when the urea concentration was about 2.0 and 5.5 mol/L.

**Fig 4:**
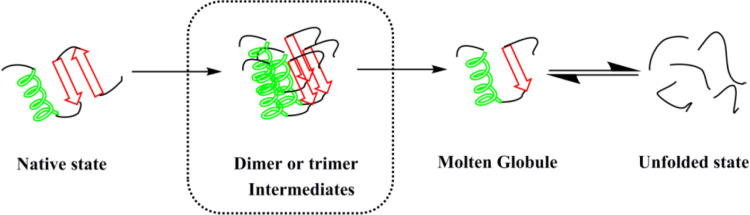
Schematic diagram of the unfolding process of SPIs and SPI-casein complexes (the intermediate in the dashed box only appears in the four-state model).

Amagliani et al. [21] believed that once the native state of the protein was disrupted, the formation of certain aggregate structures was a common feature of globular proteins, which does not depend on a specific amino acid sequence. Based on the results from literature concerning the unfolding process of globular proteins and fluorescence phase diagrams, we could speculate that there was only one intermediate, molten glubule (MG), in the three-state model of SPIs unfolding process. In the case of SPI-casein complexes, there were two intermediates, that is, dimer or trimer intermediates and MG (Fig 4).

### Surface hydrophobicity indexes of SPIs and SPIs–casein complexes during the unfolding process

To further investigate the interaction forces and possible modes between SPIs and casein, the subsequent fluorescence experiments were conducted. Surface hydrophobicity is an important parameter to measure the strength of protein intermolecular interactions, which is not only related to the processing methods and conditions but also closely related to the properties of the protein [22]. Bigelow suggested that solubility itself was influenced by two indexes, i.e., surface hydrophobicity and charge frequency, and that high charge frequency and low surface hydrophobicity could effectively improve solubility [23]. As previously mentioned, protein conformation changes continuously during urea induction, which could affect the surface hydrophobicity of protein solutions.

**Fig 5:**
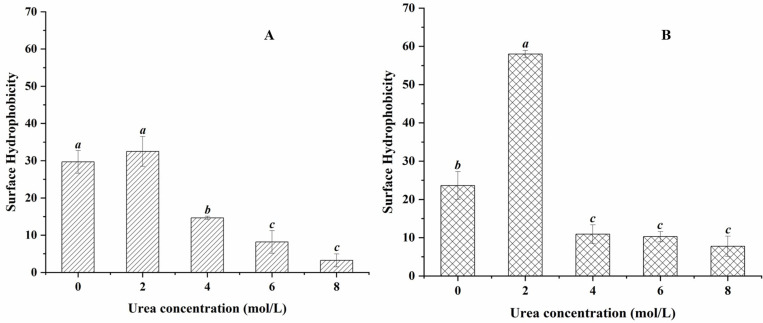
Surface hydrophobicity indexes of protein solutions treated with different urea concentrations (A: SPIs; B: SPI-casein complexes).

As shown in Fig 5A, the hydrophobicity index of SPIs decreased gradually with the increase in urea concentration, where the hydrophobicity index of urea concentration at 2 mol/L increased from 29.69 ± 3.04 to 32.49 ± 4.00 compared with that at 0 mol/L, but the difference was not significant (*p* > 0.05).

Then, the surface hydrophobicity index decreased significantly to 14.65 ±  0.46 at 4 mol/L (*p* < 0.05) and 8.17 ± 3.05 at 6 mol/L (*p* < 0.05), whereas the difference between the hydrophobicity index at 8 mol/L and 6 mol/L was not significant (*p* > 0.05). Subsequently, the surface hydrophobicity index decreased significantly to 14.65 ± 0.46 (urea concentration: 4 mol/L) (*p* < 0.05) and 8.17 ± 3.05 (urea concentration: 6 mol/L) (*p* < 0.05), whereas the difference between the hydrophobicity index at 8 mol/L urea treatment and 6 mol/L treatment was not significant (*p* > 0.05).

As for the solution of SPI-casein complexes (Fig 5B), the hydrophobicity index of the solution treated with urea at 2 mol/L increased from 23.64 ± 3.62 to 57.98 ± 0.98 compared with that at 0 mol/L, and the difference was significant (*p* < 0.05). Subsequently, the hydrophobicity index of the solution treated with urea at 4 mol/L decreased significantly to 10.92 ± 2.45 (*p* < 0.05), and the hydrophobicity index of the solution treated with urea at 6 and 8 mol/L did not differ significantly from that at 4 mol/L (*p* > 0.05).

Intermediates refer to stable conformational states with special structures formed in the process of protein unfolding or folding, with abundant secondary structures (or with a small reduction), no tertiary structures or some highly dynamic tertiary structures remaining, and dense hydrophobic centers [24–26]. The increase in the surface hydrophobicity index of SPIs and the SPI-casein complexes solution with 2 mol/L urea treatment was most likely due to the exposure of hydrophobic intermediates. At this time, both protein solutions started to be turbid. With further increase in urea concentration, the protein structures were gradually unfolded by urea, the surface hydrophobicity indexes decreased, and the two kinds of protein solutions turned clear.

### Fluorescence quenching of the unfolding process of SPIs and SPIs–casein complexes induced by urea

According to the Stern–Volmer formula, *K*_*sv*_ is the quenching constant of the quenching agent, and *f*_*m*_ is the percentage of the chromophore group in the molecule that could be approached by the quenching agent in the total fluorescent chromophore group, that is, the quenching rate [27,28]. The Stern–Volmer linear analysis indexes *K*_*sv*_ and *f*_*m*_ for SPIs at different urea concentrations are summarized in Table 1. As shown in Table 1, when the quencher acrylamide and inducer urea were present in the solution, compared with the indexes of natural SPIs, *K*_*sv*_ reached the maximum value of 4.640 ± 0.070 at 2 mol/L urea concentration and then gradually decreased to 4.003 ± 0.025 with the increase in urea concentration, but it was still significantly higher than the natural state (*p* < 0.05). By contrast, *f*_*m*_ reached more than 0.95 at 2 mol/L urea concentration, and with the increase in urea concentration, the change of this value was not significant (*p* > 0.05).

**Table 1:** Quenching parameters *K*_*sv*_ and *f*_*m*_ of acrylamide and KI as quenchers for fluorescence quenching of unfolded SPIs induced by urea at different concentrations.

Urea concentration (mol/L)	Acrylamide		Potassium iodide	
	*K*_*sv*_	*f*_*m*_	*K*_*sv*_	*f*_*m*_
0	2.253 ± 0.081c	0.573 ± 0.006b	2.160 ± 0.010c	0.385 ± 0.049c
2	4.640 ± 0.070a	0.950 ± 0.020a	5.913 ± 0.045a	0.840 ± 0.010a
4	4.383 ± 0.360ab	0.950 ± 0.069a	3.163 ± 0.035b	0.637 ± 0.025b
6	4.269 ± 0.240ab	0.989 ± 0.001a	3.158 ± 0.015b	0.662 ± 0.019b
8	4.003 ± 0.025b	0.990 ± 0.000a	3.153 ± 0.125b	0.690 ± 0.069b

Note: Different letters in the same column indicate significant difference between data (*p < *0.05).

When KI was used as the quencher, the change trend of *K*_*sv*_ was similar to that of acrylamide, reaching a maximum value of 5.913 ± 0.045 at 2 mol/L urea concentration, and *f*_*m*_ was also the highest at this time, reaching 0.840 ± 0.010; moreover, with the increase in urea concentration, the differences between *K*_*sv*_ values or *f*_*m*_ values were not significant (*p* > 0.05), and the quenching rate *f*_*m*_ was only 0.65–0.70, which was much lower than that of acrylamide under the same urea concentration treatment (above 0.95).

The Stern–Volmer linear analysis indexes *K*_*sv*_ and *f*_*m*_ for SPIs–casein complexes at different urea concentrations are summarized in Table 2, which clarified that the quenching process of the protein complexes at different urea concentrations was significantly different from that of SPIs alone. When acrylamide was the quencher, the *K*_*sv*_ value did not increase but significantly decreased to 6.240 ± 0.020 (*p < *0.05), whereas *f*_*m*_ reached above 0.95 when the urea concentration was 2 mol/L. With further increase in urea concentration, the *K*_*sv*_ value significantly increased to 7.583 ± 0.025 (*p < *0.05), and no significant changes were observed for *f*_*m*_ values. However, when KI was used as quencher, the change trend of *K*_*sv*_ value was similar to that of SPIs alone. At 2 mol/L urea concentration, the maximum value of *K*_*sv*_ was 3.983 ± 0.015, and the quenching rate *f*_*m*_ was also the highest, reaching 0.940 ± 0.087, after which the *K*_*sv*_ value decreased significantly to 2.237 ± 0.045 (*p* < 0.05), whereas *f*_*m*_ also decreased significantly to 0.063 ± 0.035 (*p* < 0.05) with increasing urea concentration.

**Table 2:** Quenching parameters *K*_*sv*_ and *f*_*m*_ of acrylamide and KI as quenchers for fluorescence quenching of unfolded SPI-casein complexes induced by urea at different concentrations.

Urea concentration (mol/L)	Acrylamide		Potassium iodide	
	*K*_*sv*_	*f*_*m*_	*K*_*sv*_	*f*_*m*_
0	8.470 ± 0.050a	0.887 ± 0.015b	2.157 ± 0.015d	0.390 ± 0.057b
2	6.240 ± 0.020e	0.980 ± 0.000a	3.983 ± 0.015a	0.940 ± 0.087a
4	6.603 ± 0.025d	0.990 ± 0.000a	3.263 ± 0.105b	0.433 ± 0.025b
6	6.972 ± 0.046c	0.990 ± 0.000a	2.545 ± 0.053c	0.371 ± 0.027b
8	7.583 ± 0.025b	0.990 ± 0.000a	2.237 ± 0.045d	0.063 ± 0.035c

Note: Different letters in the same column indicate significant difference between data (*p* < 0.05).

Acrylamide is an uncharged nonpolar quencher that could quench the fluorescence of groups on the surface or buried deep inside of the protein molecules [17,27]; whereas KI is an anionic quencher; thus, it could only quench fluorescent groups located on the surface of the protein molecule or in a polar environment close to the surface of the molecule [29,30]. The above data suggest that the fluorescent groups of SPIs, such as tryptophan residues, were exposed on the surface at a urea concentration of 2 mol/L, which allowed the two quenchers, i.e., acrylamide and KI, to play a better role in quenching. However, when SPIs was complexed with casein, the fluorescent groups of the protein complexes were also exposed in the solution at 2 mol/L urea, which makes the quenching rates of acrylamide and KI reach above 0.90; in addition, with the further increase in urea concentration, the quenching data of KI reveal that the conformation of the protein complexes changed, and the fluorescent groups in the molecule, such as tryptophan residues, were gradually embedded into the interior of the molecule, making the quencher KI unreachable, leading to the decrease in quenching rate.

### Fluorescence quenching of the unfolding process of 7S/11S and 7S/11S–casein complexes induced by urea

The quenching indexes of 7S/11S and 7S/11S–casein complexes at different urea concentrations were analyzed to preliminarily determine the interaction position of SPIs and casein. As shown in Table 3, the changing trends of *K*_*sv*_ of 11S showed high similarity to those of SPIs when the quenching agents were acrylamide and KI, both reaching the highest values at 2 mol/L urea concentrations (8.417 ± 0.059 and 4.449 ± 0.167). The *K*_*sv*_ values gradually decreased with further increase in urea concentration, but the difference was not significant (*p* > 0.05). It is worth mentioning that the *K*_*sv*_ values of 11S were significantly higher (*p* < 0.05) than that of control (urea concentration 0 mol/L) under different urea concentrations. For instance, the *K*_*sv*_ values were 3.61 and 5.71 times over the control for acrylamide and iodide quenching when exposed to 2 mol/L urea, which indicated that the tryptophan chromophore of 11S could be located on the surface as well as in the interior of the molecule. The tryptophan chromophore of 11S was continuously exposed with the increase of urea concentration, until the quenching rate *f*_*m*_ reached 0.999. The result was similar to that of Clara et al. [9] who believed that 11S unfolds in a two-step fashion and that urea treatment increased the accessibility of tryptophan chromophore. In contrast, the changing trends of 7S indexes were different from that of 11S, and the *K*_*sv*_ values at both acrylamide or KI as quenching agent gradually increased as the concentration of urea concentration increased, reaching the highest (4.960 ± 0.062 and 2.950 ± 0.061) at a urea concentration of 8 mol/L, which was significantly higher than control (*p < *0.05). However, the values were only 1.34 and 1.38 times over the control, which indicated that the tryptophan residues of 7S should be on the surface of the molecule and more accessible to quenching agents compared to 11S. The results were consistent with the findings of Deshpande [31] and Maruyama [32].

**Table 3:** Quenching parameters *K*_*sv*_ and *f*_*m*_ of acrylamide and KI as quenchers for fluorescence quenching of unfolded 7S and 11S induced by urea at different concentrations.

Urea concentration (mol/L)	7S				11S			
	Acrylamide		Potassium iodide		Acrylamide		Potassium iodide	
	*K*_*sv*_	*f*_*m*_	*K*_*sv*_	*f*_*m*_	*K*_*sv*_	*f*_*m*_	*K*_*sv*_	*f*_*m*_
0	3.697 ± 0.155d	0.9127 ± 0.020c	2.133 ± 0.156d	0.6587 ± 0.034c	2.327 ± 0.146c	0.7357 ± 0.045b	0.779 ± 0.057c	0.2573 ± 0.020c
2	4.483 ± 0.130c	0.9763 ± 0.005b	2.367 ± 0.055c	0.6837 ± 0.006bc	8.417 ± 0.059a	0.9767 ± 0.006a	4.449 ± 0.167a	0.8053 ± 0.046a
4	4.693 ± 0.075bc	0.9983 ± 0.001a	2.663 ± 0.159b	0.7213 ± 0.047b	6.783 ± 0.204b	0.9763 ± 0.009a	3.268 ± 0.210b	0.6643 ± 0.014b
6	4.717 ± 0.142b	0.9990 ± 0.000a	2.860 ± 0.078ab	0.7797 ± 0.012a	6.667 ± 0.131b	0.9870 ± 0.005a	3.226 ± 0.184b	0.6933 ± 0.026b
8	4.960 ± 0.062a	0.9990 ± 0.000a	2.950 ± 0.061a	0.7883 ± 0.006a	6.620 ± 0.089b	0.9990 ± 0.000a	3.202 ± 0.074b	0.7043 ± 0.025b

Note: Different letters in the same column indicate significant difference between data (p < 0.05).

Further analysis of the quenching indexes of 7S/11S–casein complexes were performed. As shown in Table 4, the *K*_*sv*_ values of the 11S–casein complex with acrylamide as quenching agent showed similar changing trends compared to 11S alone, i.e., the highest *K*_*sv*_ value (9.453 ± 0.215) was reached at 2 mol/L urea, after which the quenching rate *f*_*m*_ reached 0.9990 with increasing urea concentration. When KI was used as quenching agent, *K*_*sv*_ and *f*_*m*_ also varied similarly to that of 11S alone, differing only in degree, e.g., the *K*_*sv*_ value was 3.28 times over control at 2 mol/L urea concentration. In contrast, the *K*_*sv*_ values of 7S–casein complex with acrylamide as quenching agent showed different changing pattern from that of 7S. Instead of gradually increasing to the highest value for 7S, the peak values of 7S–casein complex were 7.950 ±  0.270 and 4.533 ±  0.134 at 6 and 4 mol/L urea concentrations with acrylamide and KI as quenching agent, respectively. Besides, the quenching rate did not increase with the further increase of urea concentration thereafter. It should be pointed out that when KI was used as quenching agent, the quenching rate *f*_*m*_ significantly decreased when urea concentration reached 6 mol/L, which was similar to the changes of quenching indexes of SPIs–casein complex. Thus, it could be inferred that the interaction between SPIs and casein mainly occurs in 7S.

**Table 4:** Quenching parameters *K*_*sv*_ and *f*_*m*_ of acrylamide and KI as quenchers for fluorescence quenching of unfolded 7S/11S-casein complexes induced by urea at different concentrations.

Urea concentration (mol/L)	7S-casein				11S-casein			
	Acrylamide		Potassium iodide		Acrylamide		Potassium iodide	
	*K*_*sv*_	*f*_*m*_	*K*_*sv*_	*f*_*m*_	*K*_*sv*_	*f*_*m*_	*K*_*sv*_	*f*_*m*_
0	6.827 ± 0.138c	0.8087 ± 0.0583b	3.293 ± 0.224b	0.2413 ± 0.0184c	4.283 ± 0.241c	0.8370 ± 0.068c	1.397 ± 0.064e	0.2433 ± 0.029c
2	7.417 ± 0.403b	0.8713 ± 0.0384ab	4.460 ± 0.195a	0.3893 ± 0.0132a	9.453 ± 0.215a	0.8727 ± 0.037bc	4.582 ± 0.075a	0.9147 ± 0.009a
4	7.770 ± 0.270ab	0.8920 ± 0.0353a	4.533 ± 0.134a	0.4047 ± 0.0104a	7.540 ± 0.291b	0.8820 ± 0.016bc	3.343 ± 0.214d	0.7540 ± 0.008b
6	7.950 ± 0.270a	0.9007 ± 0.0180a	3.413 ± 0.067b	0.3137 ± 0.0050b	7.640 ± 0.292b	0.9420 ± 0.026ab	3.753 ± 0.210c	0.7717 ± 0.012b
8	7.947 ± 0.055a	0.9027 ± 0.0123a	3.207 ± 0.110b	0.3157 ± 0.0061b	7.917 ± 0.180b	0.9990 ± 0.000a	4.160 ± 0.090b	0.7733 ± 0.009b

Note: Different letters in the same column indicate significant difference between data (p < 0.05).

### Molecular docking of SPIs with casein

**Fig 6:**
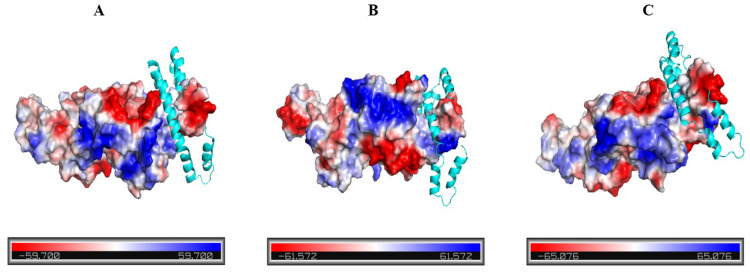
Molecular docking results of SPIs and casein (casein molecular chain presented by blue ribbon).

As inferred before, the interaction between SPIs and casein should occur mainly in 7S, thus, molecular docking of 7S chains with casein chains was conducted to speculate on the possible causes of the above phenomenon. The results of molecular docking of the three chains of 7S from SPIs (PDB:1IPK) with the α_s_-chain from casein (5WO2) are shown in Fig 6.

The 7S chain in SPIs was a qualitative electrostatic representation, whereas the casein α_s_-chain was presented as a ribbon. As shown in the figure, a pocket-like region existed in all three chains of SPIs, and the α_s_-chain of casein could enter the pocket through hydrophobic interactions and other forces to form a more stable complex. The pattern of this complex may also explain why the index changes of protein complexes were so different from that of SPIs when treated with the same urea concentration.

## Conclusions

Inhibition of amorphous aggregates formation of SPIs is often required during food processing. In this study, the possible mechanism of casein as a class of molecular chaperones to enhance the solubility of plant protein SPIs was preliminarily explored. The turbidity changing trends of SPIs/7S/11S in the presence or absence of casein indicated that casein could indeed enhance the stability of protein solutions such as SPIs. The fluorescence phase diagram results indicated that the unfolding process of SPIs-casein complexes followed a “four state” model, and that casein might have interacted with dimer or trimer intermediates, thus blocking the formation pathway of amorphous aggregates.

The quenching experiments showed that the tryptophan accessibility of 7S were higher than that of 11S, and *K*_*sv*_ and *f*_*m*_ changing trend of 7S-casein complex was similar to that of SPI-casein complex. Thus, it could be inferred that 7S might be the main interaction site between SPIs and casein. It could be seen from the molecular docking results that 7S chain could form a “pocket” conformation to “hold” the casein molecular chain. This conformation change could be the reason for casein blocking the formation of SPI amorphous aggregates.

In summary, the present study was a preliminary investigation of the mechanism of casein blocking amorphous aggregates formation of SPIs through turbidity curves and various fluorescence methods. The results could supplement the formation and regulation theory of globular protein aggregates, and provide practical guidance for the application of SPIs in the food industry and the development of foods with high content of SPIs.

## Supporting information

S1 File The raw data for turbidity and fluorometric measurements of the article were presented in the supporting files (Raw data for turbidity, fluorometric measurements etc.rar). (RAR)
